# Assessment of Medicaid Beneficiaries Included in Community Engagement Requirements in Kentucky

**DOI:** 10.1001/jamanetworkopen.2019.7209

**Published:** 2019-07-17

**Authors:** Atheendar S. Venkataramani, Elizabeth F. Bair, Erica Dixon, Kristin A. Linn, Will Ferrell, Margrethe Montgomery, Michelle K. Strollo, Kevin G. Volpp, Kristen Underhill

**Affiliations:** 1 Center for Health Incentives and Behavioral Economics, Leonard Davis Institute of Health Economics, University of Pennsylvania, Philadelphia; 2 Department of Medical Ethics and Health Policy, Perelman School of Medicine, University of Pennsylvania, Philadelphia; 3 Department of Medicine, Perelman School of Medicine, University of Pennsylvania, Philadelphia; 4 Department of Biostatistics, Epidemiology, and Informatics, Perelman School of Medicine, University of Pennsylvania, Philadelphia; 5 National Opinion Research Center (NORC) at the University of Chicago, Chicago, Illinois; 6 National Opinion Research Center (NORC) at the University of Chicago, Bethesda, Maryland; 7 Columbia Law School, New York, New York; 8 Department of Population and Family Health, Mailman School of Public Heath, Columbia University, New York, New York

## Abstract

**Importance:**

States are pursuing Section 1115 Medicaid demonstration waiver authority to apply community engagement (CE) requirements (eg, participation in work, volunteer activities, or training) to beneficiaries deemed able-bodied as a condition of coverage. Understanding the size and characteristics of the populations included in these requirements can help inform policy initiatives and anticipate effects.

**Objective:**

To estimate the number and characteristics of Kentucky Medicaid beneficiaries who would have to meet CE requirements.

**Design, Setting, and Participants:**

Cross-sectional study in which administrative records for the entire population of Medicaid beneficiaries in Kentucky as of February 2018 and original survey data, based on responses from 9396 Medicaid beneficiaries included in the waiver program, were analyzed.

**Exposures:**

Eligibility for Kentucky’s Medicaid demonstration waiver as of the originally planned implementation date (July 2018).

**Main Outcomes and Measures:**

Number of beneficiaries included in CE requirements, including those already meeting vs not meeting hour quotas and those who may qualify for medical frailty exemptions.

**Results:**

Among the 9396 individuals included in the Section 1115 waiver program who participated in the survey, the mean weighted (SD) age was 36.1 (11.9) years; a weighted 47.2% of respondents were female, and most beneficiaries (weighted percentage, 78.2%) were non-Hispanic white participants. We estimated that 132 790 (95% CI, 129 132-136 449) beneficiaries would have been required to meet CE requirements in July 2018, amounting to 40.2% of Medicaid beneficiaries included in the demonstration waiver. Of this group, 25 422 (95% CI, 23 135-27 710) beneficiaries may have qualified for a medical frailty exemption either by self-attestation (after confirmation by their Medicaid insurer) or by being identified as eligible by physicians or their insurer. Another 58 943 (95% CI, 55 687-62 196) beneficiaries likely would have met CE hour requirements and been required to report compliance. Ultimately, 48 427 (95% CI, 45 281-51 574) individuals would have had to add new activities to meet CE requirements, amounting to 14.7% of those included in the demonstration waiver as a whole and 36.3% of those included in the CE component of the waiver. Beneficiaries in the potentially medically frail group reported worse socioeconomic status, poorer health outcomes, and higher rates of hospital admission and emergency department use than those meeting CE requirements. Similarly, the group currently not meeting and not exempt from CE hour requirements reported worse socioeconomic status than those meeting the CE requirements, although magnitudes of the differences were smaller.

**Conclusions and Relevance:**

Findings suggest that most beneficiaries who would be included in CE programs either already meet activity requirements, which they will be required to proactively report, or may qualify for a medical frailty exemption. Consequently, the outcomes of CE programs will depend on states’ processes for addressing health-related, socioeconomic, and administrative barriers to participating in and reporting CE activities and identifying medical frailty.

## Key Points

**Question:**

What proportion of Medicaid beneficiaries in Kentucky would be included in community engagement (CE) requirements to maintain insurance coverage in the state’s Medicaid demonstration waiver program?

**Findings:**

In this cross-sectional study, administrative records and survey data collected at the time of the originally intended demonstration waiver implementation date (July 2018) showed that more than 130 000 beneficiaries (40.2% of those included in the demonstration waiver) would likely be required to meet CE requirements. Of this group, approximately 48 000 (14.7% of individuals included in the overall waiver and 36.3% of individuals included in CE requirements) did not meet CE hours at baseline nor were likely to qualify for medical frailty and would have had to take on new activities to maintain benefits.

**Meaning:**

The findings suggest that most beneficiaries of Medicaid in Kentucky who were included in CE requirements likely already meet required hours or qualify for an exemption for the program.

## Teaser

This cross-sectional study estimates the number and characteristics of Medicaid beneficiaries in Kentucky who would have to meet community engagement requirements under a Section 1115 Medicaid demonstration waiver.

## Introduction

In January 2018, the Centers for Medicare & Medicaid Services announced they would, for the first time, consider Section 1115 Medicaid demonstration waivers applying community engagement (CE) requirements to beneficiaries considered able-bodied.^1^ Community engagement requirements mandate that beneficiaries complete a monthly minimum of hours engaged in specified activities as a condition of receiving Medicaid benefits. Required activities vary across states but generally include paid employment, attending school or job training, seeking a job, and caregiving. Demonstration waivers with CE requirements have now been implemented in Indiana and approved in Arizona, Maine, Michigan, New Hampshire, and Wisconsin, with waiver applications containing CE requirements pending in another 8 states.^2^ Active CE requirements in Arkansas and approved CE programs in Kentucky were both recently struck down by a federal court.^3^

The rationale for making Medicaid benefits conditional on CE requirements is to motivate participation in activities that could improve health by allowing “able-bodied, working age adults to experience the dignity of a job, of contributing to their own care, and gaining a foothold on the path to independence.”^4^ However, concerns have been raised that CE requirements may lead to coverage losses if beneficiaries are unable to meet requirements owing to poor health or other life circumstances. Even if beneficiaries do meet requirements, there may be barriers to reporting compliance.^5,6,7,8^ These concerns stem from experiences with other public programs whose benefits have long been linked to work requirements.^9^ Thus, the population-level impact of Medicaid CE programs will depend on the balance between the potential salutary effects of engagement in CE activities and the potential detrimental effects of coverage losses.^10,11^

In advance of detailed evaluations of recently implemented and forthcoming Medicaid waivers, population-level estimates of potential exposure to CE requirements will be critical for projecting and tracking the health and socioeconomic effects of these waivers. While several studies have attempted to identify the population likely to be included in CE requirements, these studies have typically used economic surveys.^12,13^ Estimates of policy exposure using these surveys are limited by lags in data availability, incomplete measurement of participation in required CE activities, and limited information on health. Surveys of Medicaid beneficiaries or low-income individuals with more detailed health information address some of these gaps.^8,14,15^ However, it remains unclear whether these specific study populations accurately capture the group of individuals included in CE requirements.

This study addresses these critical gaps in the literature by using new, dedicated administrative and survey data on Medicaid beneficiaries in the Commonwealth of Kentucky. These data were collected as part of the study team’s independent evaluation of Kentucky’s demonstration waiver program, which was originally scheduled to be implemented on July 1, 2018, and then again on April 1, 2019, before the program was struck down by a federal court.^3,16^ We used these data to answer 3 research questions. First, at the originally intended time of waiver implementation, what proportion of Medicaid beneficiaries would be included in CE requirements, differentiating between those who do not meet CE hour requirements at the time of implementation, those who do meet hour quotas but would have to fulfill the administrative task of reporting their hours, and those who may be exempt from the program on the basis of medical frailty? These categories of exposure are of general interest in all 16 states applying for or already receiving Section 1115 CE waiver authority. Second, how did the share of the population included in or exempt from CE requirements vary by geographic area, which may differ markedly in available economic opportunities? Third, how did beneficiaries with different types of exposures (or exemptions) vary on socioeconomic status, health outcomes, and health care utilization?

## Methods

### Program Overview

Kentucky’s Helping to Engage and Achieve Long-Term Health (HEALTH) program is a now-remanded, previously Centers for Medicare & Medicaid Services–approved Section 1115 demonstration waiver that specifies 80 hours of monthly participation in CE. Other elements of Kentucky HEALTH include mandatory premiums tied to income, healthy behavior incentive accounts for dental and vision care, and suspension of coverage for late redetermination of eligibility.^17,18^ Participation in Kentucky HEALTH is deemed mandatory for able-bodied adults under 138% of the federal poverty line, with several exceptions for vulnerable populations (eg, former foster care youth up to age 26 years, individuals exposed to domestic violence, and refugees). Both the initial implementation of Kentucky HEALTH on July 1, 2018, and a subsequent reapproved implementation date of April 1, 2019, were blocked by a federal court decision vacating Centers for Medicare & Medicaid Services approval.

Not all individuals required to participate in Kentucky HEALTH were to be included in CE requirements. Beneficiaries automatically exempted from CE requirements included pregnant women; individuals designated medically frail based on an algorithmic assessment of administrative claims data or, for individuals new to the program or without prior claims data, clinician attestation^19,20^; individuals experiencing chronic homelessness; those already fulfilling work requirements for the Temporary Assistance for Needy Families (TANF) or Supplemental Nutrition Assistance Program (SNAP); full-time students; and primary caregivers of dependents. Beneficiaries not falling into any of these categories would be required to meet and report their monthly CE hours online, in person at a career center, over the telephone, or through a mailed form.^21^ Beneficiaries working 30 or more hours per week may have been exempt from reporting their hours on a monthly basis, although to be considered for this they would have had to proactively report meeting hour requirements to the state in advance.

Beneficiaries who were unable to participate in CE owing to health reasons but were not identified as medically frail by the state based on algorithmic assessment of medical claims data may still have been eligible for a medical frailty exemption. To obtain this exemption, the beneficiary could either complete a self-attestation form or be identified as eligible by their physician or Medicaid managed care organization (MCO). Both the beneficiary-initiated and physician-initiated processes require MCO confirmation. Nonmedically frail beneficiaries who failed to meet or report their CE hours for a month would have their Medicaid benefits suspended unless they completed those hours by the end of the next month or completed a reentry course (limited to 1 course per year), in addition to the current month’s required hours.

### Data

We used data from 2 sources. The first was administrative data comprising the entire population of Medicaid beneficiaries in Kentucky as of February 2018. The administrative data included beneficiary demographic characteristics (age, sex, race/ethnicity, geographic location) and information on whether individuals were currently pregnant, receiving long-term care services, considered medically frail based on state algorithmic assessment of medical claims, or concomitantly enrolled in TANF or SNAP.

The second data source was a survey of a random sample of beneficiaries conducted between May 5, 2018, and September 8, 2018, and fielded by the National Opinion Research Center at the University of Chicago. The survey included detailed questions on participant demographic characteristics; health care utilization; health outcomes; socioeconomic status; participation in work, volunteer activities, schooling, and caregiving; medical debt; and financial security. The sampling frame for the survey was the population flagged by the state as included in Kentucky HEALTH in the administrative data, excluding individuals 60 years and older and individuals receiving SNAP and TANF benefits. Survey weights were constructed to adjust for differential nonresponse. Further data on both data sets appears in the eAppendix in the Supplement.

The survey instrument, sampling method, and survey data collection were approved by the National Opinion Research Center institutional review board. Analysis of the collected survey data was deemed exempt by the University of Pennsylvania institutional review board, given that data analyses would be conducted using deidentified data. This report follows the Strengthening the Reporting of Observational Studies in Epidemiology (STROBE) reporting guideline for cross-sectional studies.^22^

### Statistical Analysis

**Figure 1.**
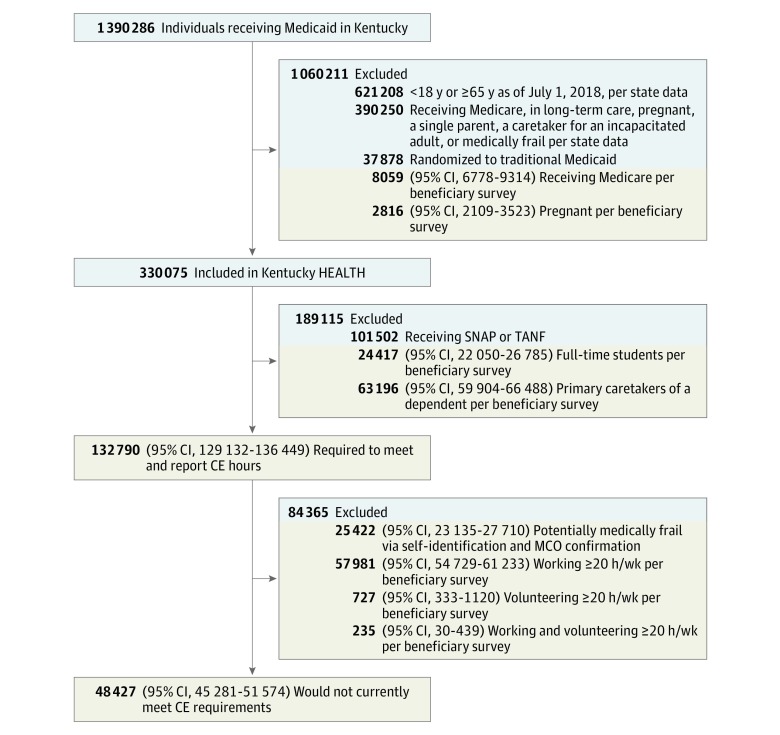
Estimates of Populations Exempt From and Included in Community Engagement (CE) Hour and Reporting Requirements Values with a blue background are exact population counts derived from administrative data. Values with a tan background are population estimates derived from the survey of Medicaid beneficiaries, which sampled from the population of residents who were exposed to Kentucky’s Helping to Engage and Achieve Long-Term Health (HEALTH) Medicaid waiver program, aged 18 to 59 years, and not receiving Supplemental Nutrition Assistance Program (SNAP) or Temporary Assistance for Needy Families (TANF). There is a discrepancy of 8170 individuals between the group included in Kentucky HEALTH and the group required to meet and report CE hours (4% of population excluded to derive population size estimates for the latter group). This difference is because the survey sampling frame did not include all individuals identified as eligible for Kentucky HEALTH (eg, the survey excluded individuals aged 60 to 64 years) and survey enumeration occurred several months after administrative data became available. See the eAppendix in the Supplement for further details on the calculations conducted. MCO indicates managed care organization.

We estimated the total number of Medicaid beneficiaries potentially included in CE requirements, with specific focus on the following mutually exclusive groups: (1) those who met CE hour requirements but would be required to report hours, (2) those who were initially identified as included in CE hour requirements but may qualify for a medical frailty exemption through self-identification and Medicaid MCO confirmation, and (3) those who did not meet the CE hour requirements (Figure 1). From the total population of Medicaid beneficiaries, we first excluded individuals who were exempt from the Kentucky HEALTH waiver program as a whole, including individuals receiving long-term care (administrative data), individuals who were deemed medically frail by proprietary state algorithms that use claims data (administrative data), individuals who were pregnant (administrative and survey data), beneficiaries receiving Medicare (administrative and survey data), single parents (administrative data), individuals caring for incapacitated adults (administrative data), and individuals who were randomized by the state to the control group as part of the demonstration waiver evaluation plan.^16^ While receipt of Medicare and current pregnancy are flagged in the administrative data, we further excluded individuals reporting either in the survey to capture changes in status between the time of administrative data collection and survey data collection.

From this group of waiver-exposed individuals, we excluded beneficiaries participating in SNAP and TANF (based on administrative records) and those engaged as a primary caregiver of a dependent (survey data). Both sets of beneficiaries would be exempted from Medicaid CE requirements (SNAP and TANF beneficiaries must complete work requirements for those programs), although they would remain exposed to other elements of the waiver (such as payment of premiums, requirements for timely redetermination, and suspensions and/or lockouts for not meeting requirements).

Among remaining beneficiaries (ie, those potentially included in CE requirements), we estimated the number of beneficiaries who potentially qualified for medical frailty exemptions by self-identification and MCO confirmation. These were individuals who were not identified as medically frail according to the state algorithm but who self-reported health conditions that may interfere with CE activities before or after program implementation. We identified this population using survey data from respondents who reported having a serious or complex health condition and 1 or more days of activity-limiting poor physical or mental health in the past 30 days.^23^

From the remaining pool of beneficiaries, we assigned individuals who reported spending at least 20 hours per week engaged in work, volunteering, or caretaking as currently meeting CE hour requirements. Under Kentucky HEALTH, these individuals would meet CE hour requirements but would have to report their hours using the monthly reporting mechanisms. This group includes individuals working 30 or more hours a week who can gain an exemption from the CE requirement by proactively reporting that they may already meet requirements. Beneficiaries spending less than 20 hours per week engaged in these activities were considered not meeting CE hour requirements. This group would need to increase their CE activities, and they would also be required to report CE hours. Among this group, we estimated the number of individuals reporting having looked for work in the last 4 weeks, as job search activities can contribute to CE requirements. However, we did not designate these individuals as meeting CE requirements as we did not have information on hours spent on job search activities.

To assess spatial differences in exposure to CE requirements, we conducted the above exercise for each of the 10 local workforce development areas in Kentucky, which reflect areas with distinct socioeconomic profiles that guide state and local investment in employment generation activities (eTable in the Supplement).^24^ Community engagement requirements in the Kentucky HEALTH program would have been implemented with input from local workforce boards, who would assist in providing beneficiaries with information on available job, volunteer, and educational opportunities.

Last, we assessed socioeconomic, health, and health care utilization characteristics of beneficiaries currently meeting CE requirements and required to report their hours, beneficiaries who may be able to access medical frailty exemptions but who were not automatically excluded from CE, and beneficiaries not meeting CE requirements. For socioeconomic characteristics, we estimated proportions who are currently employed,^25^ have access to internet at home or elsewhere, and report having medical debt.^25,26^ For health measures, we examined validated measures of poor or fair physical and mental health (based on 5-point Likert scales), the number of poor mental and physical health days in the last 30 days,^27^ and the proportion of beneficiaries reporting at least 1 day of their usual activities being limited by a health condition in the last 30 days.^23^ We also examined a measure of health literacy,^28^ given its importance in explaining disparities in health care access and outcomes.^29,30,31,32^ For health care utilization, we estimated the proportion of beneficiaries who had a usual source of care and needed any medical care in the last 6 months.^25^

We used the jackknife method to account for complex survey design in all calculations using survey data. The standard errors from prediction of proportions in the survey data were used to calculate 95% CIs. All analyses were conducted using SAS version 9.4 (SAS Institute), R version 3.5.2 (The R Foundation), and Stata version 15.1 (StataCorp).

## Results

Figure 1 presents a flow diagram with estimates of the population of beneficiaries potentially exposed to the Kentucky HEALTH demonstration waiver and estimates of the number of beneficiaries exempt from or included in CE requirements. As of February 8, 2018 (the date of our administrative data extract), there were nearly 1.4 million Medicaid beneficiaries in Kentucky. Overall, 9396 individuals completed the survey, resulting in a yield rate of 16.7% (and a response rate of 29.1%, as conservatively calculated using the American Association of Public Opinion Research definition 1^33^), comparable with other surveys of Medicaid beneficiaries and low-income populations more generally.^34,35,36,37^ Among the individuals included in the Section 1115 waiver program who participated in our survey, the mean (SD) age was 36.1 (11.9) years; a weighted 47.2% of respondents were female, and most beneficiaries (weighted percentage, 78.2%) were non-Hispanic white.

After accounting for individuals outside the program’s age eligibility criteria, those meeting standard exemptions (including pregnancy, Medicare status, single parent status, caring for an incapacitated adult, and medical frailty determined by a Medicaid claims–based administrative algorithm) either at the time of the administrative data extract or of the survey several months later, and those randomized to the traditional Medicaid control group who would have otherwise been eligible, 330 075 beneficiaries would have been included in the demonstration waiver program at the time of the originally planned implementation.

Within this population, 101 052 were already obliged to meet work requirements for the SNAP or TANF programs and thus would have been exempt from the Medicaid CE requirements. An estimated 24 417 (95% CI, 22 050-26 785) and 63 196 (95% CI, 59 904-66 488) beneficiaries were not required to participate because they were full-time students or primary caregivers of a dependent, respectively.

We estimated that 132 790 (95% CI, 129 132-136 449) beneficiaries, ie, 40.2% of those exposed to Kentucky HEALTH, would likely have been included in CE hour and reporting requirements. Of this group, 25 422 (95% CI, 23 135-27 710) may have been eligible for a medical frailty exemption, either by self-attestation (with MCO confirmation) or through identification by physicians and MCOs. Among remaining beneficiaries, 58 943 (95% CI, 55 687-62 196) were engaged in activities that met CE requirements but would be required to report hours or could qualify for an exemption from monthly reporting if they proactively informed the state that they were already working 30 or more hours per week.

Excluding medically frail individuals, we estimated that 48 427 (95% CI, 45 281-51 574) did not meet CE requirements. This group represented 36.3% of the population included in Medicaid CE requirements before the originally planned implementation, 14.7% of the population exposed to the Kentucky HEALTH demonstration program as a whole at the same time point, and 3.5% of Kentucky’s total Medicaid population as of February 2018. Among these individuals not meeting CE requirements and likely ineligible for medically frailty exemptions, 24 963 (95% CI, 22 715-27 212) reported searching for work in the 4 weeks before the survey, suggesting that this estimate may be an overcount. However, we could not estimate whether or not these job-seeking activities would fulfill CE hour quotas. In addition, among those eligible for medical frailty exemptions, 17 572 (95% CI, 15 424-18 637) would not have met CE hour requirements. Thus, in the event that medical frailty exemptions were difficult to obtain for accessibility or administrative reasons, our estimate of the population not meeting CE hour requirements may be an undercount.

**Figure 2.**
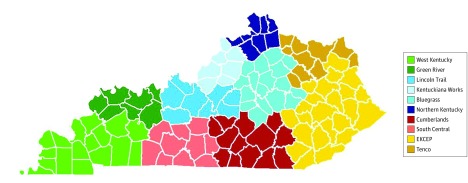
Kentucky Local Workforce Areas Map depicts Kentucky’s 10 local workforce areas, which are delineated based on unique economic and sociodemographic characteristics. EKCEP indicates Eastern Kentucky Concentrated Employment Program.

Table 1 presents estimates of the population of beneficiaries included in Kentucky HEALTH and CE requirements by local workforce areas (Figure 2). The largest numbers of individuals included in Kentucky HEALTH were in Kentuckiana Works, the largest workforce area by population (which includes Louisville) and the Eastern Kentucky Concentrated Employment Program, which has the highest unemployment rate and lowest rate of labor force participation (eTable in the Supplement). Conditional on the number of beneficiaries included in the demonstration waiver program, the estimated proportions of potentially exempt individuals owing to self-identified medical frailty, meeting and required to report CE hours, and not currently meeting CE hour requirements were relatively similar across the local workforce areas.

**Table 1.** Estimates of Populations Exempt From and Included in CE Reporting and Hour Requirements by Local Workforce Areas

Local Workforce Area^a^	Overall Kentucky HEALTH–Eligible Population^b^	No. (%) [95% CI]		
		Potentially Medically Frail With Self-identification and Managed Care Organization Confirmation	Required to Report and Would Meet CE Hours^c^	Required to Report and Would Not Meet CE Hours^d^
West				
Green River	14 637	1282 (8.8) [634-1930]	2569 (17.6) [1414-3723]	3130 (21.4) [1784-4477]
West Kentucky	27 635	2281 (8.3) [1440-3121]	4849 (17.5) [3240-6457]	3745 (13.6) [2526-4965]
Central				
Bluegrass	49 705	5016 (10.1) [3681-6351]	10 481 (21.1) [8231-12 731]	6255 (12.6) [4846-7664]
Kentuckiana Works	59 409	5665 (9.5) [4350-6980]	14 052 (23.7) [11 633-16 470]	12 080 (20.3) [9731-14 428]
Lincoln Trail	17 215	1273 (7.4) [631-1914]	3876 (22.5) [2516-5236]	2616 (15.2) [1652-3579]
Northern Kentucky	22 245	2399 (10.8) [1357-3442]	4704 (21.1) [3083-6325]	3462 (15.6) [2189-4734]
South				
Cumberlands	30 365	2072 (6.8) [1326-2817]	5507 (18.1) [3754-7260]	3989 (13.1) [2644-5334]
South Central	21 922	1696 (7.7) [1042-2350]	4399 (20.1) [2927-5870]	2763 (12.6) [1813-3713]
East				
EKCEP	51 387	2853 (5.6) [1903-3802]	5896 (11.5) [4322-7470]	7724 (15.0) [5400-10 048]
Tenco	17 260	890 (5.2) [398-1383]	2615 (15.2) [1542-3688]	2667 (15.5) [1641-3694]

Abbreviations: CE, community engagement; EKCEP, Eastern Kentucky Concentrated Employment Program; Kentucky HEALTH, Kentucky’s Helping to Engage and Achieve Long-Term Health.

^a^ See Figure 2 for a map of local workforce areas.

^b^ Overall population excludes the control population, suspended individuals, individuals with invalid telephone numbers, individuals younger than 18 years or at least 60 years, individuals receiving Medicare, individuals in long-term care, individuals administratively designated medically frail, and pregnant women.

^c^ Includes individuals involved in at least 20 hours per week of working, volunteering, and caretaking.

^d^ Includes individuals engaged in less than 20 hours per week of working, volunteering, and caretaking. Individuals who engaged in job-seeking activities in the week before the survey were not excluded.

Demographic, socioeconomic, and health characteristics and measures of health care utilization for groups of beneficiaries by type of exposure to CE are presented in Table 2. Compared with beneficiaries meeting CE requirements, beneficiaries who were deemed possibly meeting criteria for self-identified, MCO-confirmed exemption for medical frailty were on average older (mean [SD] age, 36.7 [12.6] years vs 40.3 [11.9] years, respectively) and more likely to be non-Hispanic white (1062 [75.2%] vs 503 [80.1%], respectively). They were also more likely than beneficiaries meeting CE requirements to have reported fair or poor physical or mental health (physical: 416 [64.8%] vs 236 [16.1%]; mental: 389 [60.7%] vs 259 [16.9%]), reported a greater number of days in the last month with poor physical or mental health (physical: mean [SD], 15.1 [10.9] vs 3.7 [7.2] days; mental: 13.6 [10.9] vs 3.4 [6.8] days), and reported poor health literacy (66 [11.5%] vs 58 [3.8%]). These individuals also reported greater health care needs, with greater frequencies of hospitalization and emergency department use in the last 6 months. Finally, potentially medically frail individuals were more likely than the group meeting CE requirements to have not completed high school (95 [14.6%] vs 110 [8.0%]), were less likely to be employed (240 [35.1%] vs 1398 [98.3%]), and were more likely to currently report medical debt (258 [40.6%] vs 362 [25.7%]).

**Table 2.** Demographic, Self-reported Health, Health Care Utilization, and Financial Characteristics of 3 Kentucky Helping to Engage and Achieve Long-Term Health (HEALTH) CE Populations

Survey Question	No. (%)		
	Potentially Medically Frail, Pending Self-identification and MCO Confirmation	Required to Report and Would Meet CE Hours^a^	Required to Report but Would Not Meet CE Hours^b^
Unweighted sample size	629	1424	1074
Weighted population	25 422	58 941	48 427
**Demographic Characteristic**			
Age, mean (SD), y	40.3 (11.9)	36.7 (12.6)	37.4 (12.6)
Women	388 (52.1)	756 (43.9)	548 (39.0)
Race/ethnicity^c^			
Non-Hispanic white	503 (80.1)	1062 (75.2)	841 (79.3)
Non-Hispanic black	59 (9.4)	197 (13.0)	131 (11.2)
Hispanic	13 (1.5)	76 (5.3)	37 (3.9)
Other	49 (8.2)	75 (5.6)	52 (4.5)
Marital status^c^			
Married	143 (19.4)	351 (23.0)	256 (21.1)
Other	481 (79.8)	1067 (76.5)	813 (78.2)
**Health**			
Fair or poor physical health	416 (64.8)	236 (16.1)	293 (27.1)
Poor physical health in the last 30 d, mean (SD), d	15.1 (10.9)	3.7 (7.2)	5.6 (8.7)
Fair or poor mental health	389 (60.7)	259 (16.9)	287 (25.3)
Poor mental health in the last 30 d, mean (SD), d	13.6 (10.9)	3.4 (6.8)	4.8 (8.2)
≥1 d Where usual activities were limited by a health condition in last 30 d	629 (100)	354 (25.7)	362 (33.3)
Poor health literacy^d^	66 (11.5)	58 (3.8)	64 (5.9)
**Health Care Utilization**			
Have a usual source of care	569 (90.1)	1186 (82.3)	879 (79.1)
Needed medical care in the last 6 mo	561 (88.0)	880 (61.2)	671 (60.1)
ED utilization in the last 6 mo^c^			
0	394 (62.3)	1091 (76.2)	754 (69.7)
≥1	220 (35.4)	310 (22.2)	287 (26.6)
Times admitted to the hospital in the last 6 mo^c^			
0	547 (87.9)	1345 (94.1)	981 (91.4)
≥1	78 (11.4)	68 (4.8)	78 (7.1)
**Socioeconomic Characteristics**			
Education^c^			
<High school	95 (14.6)	110 (8.0)	172 (15.4)
High school	322 (54.2)	762 (52.6)	606 (56.1)
≥Some college	209 (30.7)	545 (39.0)	280 (27.2)
Employed	240 (35.1)	1398 (98.3)	188 (17.5)
Internet access			
Home	545 (85.6)	1297 (90.8)	870 (81.3)
Home or elsewhere	584 (91.9)	1341 (94.0)	943 (88.2)
Currently owe debt for medical expenses	258 (40.6)	362 (25.7)	219 (19.8)

Abbreviations: CE, community engagement; ED, emergency department; MCO, managed care organization.

^a^ Includes individuals involved in at least 20 hours per week of working, volunteering, and caretaking.

^b^ Includes individuals engaged in less than 20 hours per week of working, volunteering, and caretaking.

^c^ Subcategories may not sum to 100% because of “don’t know” and “skip/refused” responses.

^d^ Defined as a response of often or always to the question “How often do you need to have someone help you when you read instructions, pamphlets, or other written material from a doctor, another health care provider, or a pharmacy?”

Like the potentially medically frail group, those not meeting CE requirements generally had worse socioeconomic status and health status and higher rates of hospital and emergency department use in the last 6 months compared with those meeting CE requirements. However, the magnitudes of these differences were smaller. Rates of internet access at home or any location, respectively, ranged from 870 (81.3%) and 943 (88.2%) for those required to report but not meeting CE requirements compared with 1287 (90.8%) to 1341 (94.0%) for those required to report and meeting CE requirements.

## Discussion

In this study of Medicaid beneficiaries in Kentucky, we found that 14.7% of beneficiaries who were eligible to be enrolled in the multicomponent Medicaid demonstration waiver (36.3% of beneficiaries who would have been included in the CE reporting component specifically) likely did not meet required CE hours at the time of originally scheduled implementation in July 2018. A larger number of beneficiaries were either meeting CE requirements at the time of interview (but would still have been required to report their participation) or potentially eligible for a self-attested, physician-initiated, or MCO-initiated medical frailty exemption. The proportion of beneficiaries included in CE requirements did not vary by geographic area despite large differences in economic opportunities across Kentucky workforce areas. Socioeconomic and health outcome characteristics varied across these differently exposed groups, with the poorest outcomes for the medically frail group (of whom only approximately 30% would meet CE hour requirements in the event they did not obtain the exemption) followed by the group not meeting CE hour requirements. Finally, most (approximately 60%) beneficiaries who would have been included in overall demonstration waiver requirements at the time of intended implementation were exempt from the CE component entirely owing to concomitant participation in SNAP and TANF, enrollment as a full-time student, or serving as the primary caregiver for a dependent.

Our findings illustrate the utility of combining dedicated administrative and survey data to identify different types of exposure to CE requirements, which is critical for designing programs and predicting effects. To our knowledge, this method had not been possible in prior work.^8,12,13,14,15^ For example, a 2018 article using data from the 2014 Survey of Income and Program Participation estimated that approximately 28 000 potentially exposed able-bodied individuals did not meet CE requirements at the time.^13^ Our estimate of the number of beneficiaries not currently meeting CE requirements was nearly twice as large even after explicitly accounting for individuals who may self-identify as potentially medically frail. The difference in estimates may owe to our use of more recent data, which included granular information on hours spent on most types of CE activities.

Our findings also reveal several insights for Medicaid demonstration waiver programs seeking to implement CE requirements. First, ensuring a seamless process for applying for and adjudicating medical frailty will be critical if states seek high accuracy in identifying the intended population of adults considered able-bodied. States will likely have processes in place to identify medically frail individuals through claims data, but we identified a number of people not detected by claims data who self-reported possible medical frailty. If these individuals were all unsuccessful in obtaining medical frailty exemptions, our data suggest that most (approximately 69% or >17 500 individuals) would be required to undertake new activities to meet CE hours. At present, states with approved or proposed CE waivers have proposed a variety of strategies for ex post determination of medical frailty, although the accessibility and effectiveness of these different strategies in ensuring that potentially eligible individuals can successfully obtain exemptions remain unknown.^5^

Second, for participants required to meet CE requirements or those who would otherwise have to obtain attestation of exemptions owing to medical frailty or a change in status (eg, pregnancy, receipt of SNAP or TANF, becoming a caregiver for a dependent), important program barriers could impede reporting despite CE hours compliance. For example, among the beneficiaries potentially eligible for medical frailty exemptions, we found higher rates of poor health literacy, lower rates of education, and worse physical and mental health, all of which may prevent timely self-identification and assessment by a health care professional to obtain exemptions or reporting compliance with CE hour requirements. In our study population, access to the internet at home was not universal, suggesting that between 10% and 20% of eligible beneficiaries may need to report CE hours through other means, such as telephone or mail. The administrative burdens of reporting may be more significant for this group. Moreover, health-related, socioeconomic, and administrative challenges to meeting and/or reporting CE requirements may additionally vary by geography, given stark regional differences in socioeconomic opportunities within states.

Third, the composition of the population required to report CE activities will also depend on states’ infrastructure for automating exemptions and/or compliance monitoring. States could ease administrative burdens by designing benefits systems that integrate Medicaid eligibility data with other existing administrative sources, specifically information from unemployment insurance benefits or compliance with work requirements in other benefits programs (eg, SNAP or TANF). Systems that ex ante automatically verify compliance or exempt individuals could greatly ease administrative burdens for beneficiaries compared with systems that require beneficiary or third-party reporting. For example, in Kentucky, more than 101 000 individuals were automatically exempt from CE reporting requirements owing to participation in SNAP and TANF, representing 31% of all individuals who would be included in overall demonstration waiver requirements.

### Limitations

There are several limitations to this study. First, the results represent a snapshot of the Kentucky Medicaid population from 2018, on the eve of the original implementation date of the demonstration waiver. While these data are more recent than those used in related work,^12,13,14^ it is possible that the population that would be included in CE requirements at a future implementation date may differ in size and demographic, socioeconomic, and health characteristics.

Second, our analysis relies on data from a large, original, population-based survey, which may be prone to bias from social desirability and differences between responders and nonresponders. We sought to reduce social desirability bias by ensuring confidentiality of patient responses and making clear that the research survey was independent from the state’s Medicaid program. We sought to reduce bias from nonresponse by weighting our data using sociodemographic characteristics for the universe of beneficiaries from which respondents were sampled.

Third, identification of some population groups, such as those who may be eligible for self-attested, MCO-confirmed medical frailty exemptions, is hindered by the inherent difficulty of assessing and identifying disability and medical frailty in survey data. We were unable to determine whether individuals who reported looking for work in the last month would meet CE hour requirements with job search activities. We were also unable to identify some populations, such as individuals living in the 8 rural counties that compose the Highlands Federal Promise Zone, who were exempted from CE requirements owing to concomitant federal investment targeting this region’s high unemployment rates. The study may therefore overestimate the number of beneficiaries not currently meeting CE requirements, which would strengthen the conclusion that a fairly small share of the beneficiary population would need to add new activities to comply with CE.

Fourth, findings from Kentucky may not apply to other states whose Medicaid populations and proposed demonstration waivers are distinct. Nevertheless, the broad findings from these data may be informative for general program design, particularly given the similarities in CE requirements across states.

Fifth, our estimates do not account for exposure to other elements of the demonstration waiver, such as cost sharing, requirements for timely redetermination, and health behavior incentives; these elements of Kentucky HEALTH were intended to complement CE requirements. The overlap in these program elements and the effects of these program components will require specific consideration in future work.

## Conclusions

The findings of this study suggest that most Medicaid beneficiaries who would be included in CE programs either already meet activity requirements, which they will be required to proactively report, or qualify for a medical frailty exemption. Consequently, CE program outcomes will depend on states’ processes to address health-related, socioeconomic, and administrative barriers to participating in and reporting CE activities and identifying medical frailty.

## Notes

Supplement. **eAppendix.** eMethods **eTable. **Characteristics of Local Workforce Areas in Kentucky
