# Supplementary material for: Assessment of Medicaid Beneficiaries Included in Community Engagement Requirements in Kentucky
Source: JAMA Netw Open. 2019 Jul 17;2(7):e197209. doi: 10.1001/jamanetworkopen.2019.7209 (PMC6647552; doi:10.1001/jamanetworkopen.2019.7209)
Supplement: Supplement. — eAppendix. eMethods eTable. Characteristics of Local Workforce Areas in Kentucky [file jamanetwopen-2-e197209-s001.pdf]

Supplementary Online Content

Venkataramani AS, Bair EF, Dixon E, et al. Assessment of Medicaid beneficiaries included in community engagement requirements in Kentucky. *JAMA Netw Open*. 2019;2(7):e197209. doi:10.1001/jamanetworkopen.2019.7209

eAppendix. eMethods
eTable. Characteristics of Local Workforce Areas in Kentucky

This supplementary material has been provided by the authors to give readers additional information about their work.

© 2019 Venkataramani AS et al. *JAMA Network Open.*

eAppendix. eMethods

1. Additional Information on Data Sources

We used data from two sources. The first was administrative data comprising the entire population of Medicaid beneficiaries in the Commonwealth as of Feb 8, 2018 (obtained from the Kentucky Cabinet for Health and Family Services, Department for Medicaid Services). The data were extracted on this date to allow the state sufficient time to randomize beneficiaries for evaluation purposes (given that the principal mode of evaluation of the demonstration waiver was designed to be a randomized controlled trial), and to allow the research team to collect baseline survey data prior to the originally planned implementation date (July 1, 2018). The administrative data included beneficiary demographics (age, gender, race/ethnicity, geographic location) and information on whether individuals were currently pregnant, receiving long-term care services, medically frail based on state algorithmic assessment of medical claims, or concomitantly enrolled in TANF or SNAP.

The second data source was a survey of a random sample of beneficiaries between May 5-September 8, 2018 fielded by the National Opinion Research Center at the University of Chicago (NORC). The survey was designed to obtain baseline data for the study team's five-year evaluation of the Kentucky HEALTH program. The survey included detailed questions on participant demographics; health care utilization; health outcomes; socioeconomic status; participation in work, volunteer activities, schooling, and caregiving; and medical debt and financial security. The sampling frame for the survey (from which representative survey weights were created) was the population flagged by the state as subject to Kentucky HEALTH in the above Medicaid administrative data extract, excluding individuals ages 60 and up (who would age into Medicare by the end of the 5-year study period) and individuals on SNAP and TANF; program-eligible individuals in these categories were randomized and followed in administrative data, but excluded from the survey. The survey followed a two-stage sampling strategy. First, census tracts were randomly sampled based on probability proportional to size. Second, a random sample of those randomized to the Kentucky HEALTH treatment and all individuals randomized to the traditional Medicaid control group were asked to participate in the survey. Beneficiaries were invited to take the survey by web or phone and were provided a small gift ($25 cash) for their participation. In total, 9,396 individuals completed the survey. The yield rate for the survey was 16.7%. Response rates using the different definitions put forth by the American Association of Public Opinion Research (see https://www.aapor.org/AAPOR_Main/media/MainSiteFiles/FindingE.pdf) were as follows: 29.1% (definition 1), 30.4% (definition 2), 46.8% (definition 3), and 48.9% (definition 4). These response rates are comparable to other surveys of Medicaid beneficiaries and low-income populations, more generally.[21-24] Survey weights were constructed using know sociodemographic characteristics of the universe of beneficiaries eligible for the survey study adjust for item non-response.

2. Additional Information on Calculations of Numbers of Beneficiaries Exposed to CE Hour Requirements

To identify the number of beneficiaries eligible for the Section 1115 Demonstration waiver program, we first used the administrative data to account for key excluded populations,

© 2019 Venkataramani AS et al. *JAMA Network Open.*

specifically children under the age of 18 and adults age 65 years or older, individuals on long term care, individuals who are pregnant (at the time of the data extract), single parents, individuals caring for incapacitated adults, and those identified as medically frail by a proprietary state algorithm based on existing administrative claims data and, for those new to the programs or without a prior claims history, clinician attestation. The algorithm categorizes medical conditions by severity, using first broad clinical criteria and then historical pharmaceutical and cost data. Beneficiaries then receive a score which sums the severity of all reported conditions. Individuals are deemed medically frail and automatically exempted if their score exceeds a certain threshold (which is also proprietary). Further details can be found here: https://www.wakely.com/sites/default/files/files/content/identifyingmedicallyfrailpopulationsakentuckymedicaidcasestudy20180418-6.pdf. We additionally used survey data to identify any new individuals who gained Medicare coverage or became pregnant between the period of the administrative data extract and survey enumeration. We also excluded individuals who would have been subject to demonstration waiver requirements, but were randomized by the state to a control group slated to receive traditional Medicaid as part of our independent evaluation analysis plan.

Among beneficiaries who were subject to the Kentucky HEALTH demonstration waiver, we then excluded populations who were, in most cases, automatically exempt from reporting meeting or reporting community engagement requirements. We used the administrative data to exclude participants who were receiving SNAP and/or TANF benefits as of February 8, 2018, as this group had a separate work requirement they were required to complete (which were to supercede Medicaid CE requirements). We also excluded, using survey data, two groups who could opt-in to CE requirements, full time students and primary caregiver of a child or incapacitated adult dependent. Dependents were defined as anyone responding "yes" to the question, "Are you responsible for the care of a child or an incapacitated member of your household?"

Because of data limitations, we were unable to exclude individuals living in the eight counties that make up the federally designated Kentucky Highlands Promise Zone (Bell, Clay, Harlan, Knox, Leslie, Letcher, Perry, and Whitley). Beneficiaries living in these areas were exempt on the account of historically low employment rates and federal assistance to fund efforts to improve economic opportunities in these areas. We were also unable to exclude former foster youth (up to age 26), for whom participation in CE was deemed optional. Lastly, because our survey sampling frame excluded some populations who could be eligible for CE (e.g., individuals above age 60, who were excluded as they would age out of the program over a 5 year demonstration project for which we intended to collect longitudinal data), as well as the time interval between the availability of administrative data and our survey data collection efforts, up to 8200 individuals (<2.5% of the population eligible for Kentucky HEALTH) may have been excluded from our count of beneficiaries subject to CE hour and reporting requirements. We posited that any bias created by any of these limitations would be small, given the relatively small fraction of individuals in each group.

Finally, we used survey data to subdivide the population of beneficiaries subject to CE requirements into (1) those who might qualify for medical frailty via a separate process involving either self-attestation (with managed care organization confirmation) or identification by a physician or managed care organization official, (2) those who met CE hours but were required

© 2019 Venkataramani AS et al. *JAMA Network Open.*

to report it, and (3) those who were not meeting CE hours. We started by accounting for potentially medical frail individuals, given the policy relevance of determining frailty and the reporting burden obtaining this exemption may confer. We defined of individuals who potentially qualified for an exemption … who answered "Yes" to the question "Thinking about [these conditions/this condition], as well as any other conditions you have, do you consider yourself to have any health conditions that are serious or complex? / Do you consider yourself to have any health conditions that are serious or complex?" and ≥1 day to the question "During the past 30 days, for about how many days did poor physical or mental health keep you from doing your usual activities, such as self-care, work, or recreation? Your best estimate is fine."

In differentiating between those meeting versus not meeting CE hours, our survey data did not include information on hours spent job searching, though participation in this activity was recorded. Consequently, we assigned these individuals as not currently meeting CE hours, though it is possible that some of them may have.

© 2019 Venkataramani AS et al. *JAMA Network Open.*

eTable. Characteristics of Local Workforce Areas in Kentucky

Region	**West**		**Central**				**South**		**East**	
Workforce Area	Green River	West Kentucky	Bluegrass	Kentuckiana Works	Lincoln Trail	Northern Kentucky	Cumberlands	South Central	EKCEP	Tenco
**Female** [a]	50.2%	49.3%	50.6%	50.8%	49.5%	50.0%	50.1%	50.4%	49.6%	50.3%
**Race**										
Non-Hispanic white	89.2%	85.0%	82.5%	73.5%	85.3%	90.2%	94.3%	86.6%	96.4%	94.4%
Non-Hispanic black or African American	4.9%	8.7%	8.4%	16.8%	6.9%	3.3%	1.7%	6.2%	1.4%	2.0%
Hispanic	2.8%	3.3%	4.6%	4.7%	3.7%	3.1%	2.1%	3.5%	0.9%	1.5%
Non-Hispanic other	3.1%	3.0%	4.6%	4.9%	4.1%	3.4%	2.0%	3.7%	1.4%	2.1%
**Education** [b]										
<High school	13.7%	14.8%	12.5%	10.6%	13.2%	10.6%	21.7%	16.7%	26.7%	16.0%
High School	38.0%	36.4%	28.7%	28.7%	36.6%	31.3%	38.9%	36.2%	37.2%	36.2%
Some college or more	48.3%	48.7%	58.7%	60.7%	50.2%	58.1%	39.4%	47.0%	36.1%	47.7%
**Labor force participation** [c]	59.1%	56.2%	63.9%	65.6%	61.6%	66.4%	50.9%	59.2%	41.7%	52.7%
**Employed** [d]	55.3%	50.2%	59.7%	61.5%	55.4%	62.7%	46.4%	55.4%	37.2%	48.6%
**Living in poverty** [e]	17.0%	17.8%	17.7%	13.0%	14.3%	11.2%	24.4%	18.5%	30.3%	20.6%
**Uninsured**	6.4%	8.9%	8.2%	6.9%	7.5%	6.8%	8.7%	8.8%	8.7%	8.9%

Note: Data from Social Explorer American Community Survey (ACS), 5-year estimates (2013-2017). Subgroups may add to more than 100% due to rounding. See Figure 2 of the main text for a map of Kentucky local workforce areas.

[a] Among 18-64 year olds

[b] Among individuals aged ≥25

© 2019 Venkataramani AS et al. *JAMA Network Open.*

[c] Among individuals aged ≥16
[d] Among civilians aged ≥16
[e] Among 18-64 year olds for whom poverty status was determined

© 2019 Venkataramani AS et al. *JAMA Network Open.*
